# Spatial Analysis of Built Environment Risk for Respiratory Health and Its Implication for Urban Planning: A Case Study of Shanghai

**DOI:** 10.3390/ijerph16081455

**Published:** 2019-04-24

**Authors:** Lan Wang, Wenyao Sun, Kaichen Zhou, Minlu Zhang, Pingping Bao

**Affiliations:** 1 College of Architecture and Urban Planning, Tongji University, 1239 Siping Road, Shanghai 200092, China; 1630043@tongji.edu.cn (W.S.); 1730042@tongji.edu.cn (K.Z.); 2 Shanghai Center for Disease Prevention and Control, Shanghai 200336, China; zhangminlu@scdc.sh.cn

**Keywords:** built environment, respiratory health, spatial analysis, geographical detector, urban planning

## Abstract

Urban planning has been proven and is expected to promote public health by improving the built environment. With a focus on respiratory health, this paper explores the impact of the built environment on the incidence of lung cancer and its planning implications. While the occurrence of lung cancer is a complicated and cumulative process, it would be valuable to discover the potential risks of the built environment. Based on the data of 52,009 lung cancer cases in Shanghai, China from 2009 to 2013, this paper adopts spatial analytical methods to unravel the spatial distribution of lung cancer cases. With the assistance of geographic information system and Geo-Detector, this paper identifies certain built environments that are correlated with the distribution pattern of lung cancer cases in Shanghai, including the percentage of industrial land (which explains 28% of the cases), location factors (11%), and the percentages of cultivated land and green space (6% and 5%, respectively). Based on the quantitative study, this paper facilitates additional consideration and planning intervention measures for respiratory health such as green buffering. It is an ecological study to illustrate correlation that provides approaches for further study to unravel the causality of disease incidence and the built environment.

## 1. Introduction

Lung cancer has become a serious public health problem throughout the world. According to data provided by the International Agency for Research on Cancer (IARC) in 2018, the incidence and mortality of lung cancer ranked first among all cancers and the total number of new lung cancer cases worldwide was about 2.09 million, accounting for 11.6% of all new cancer cases, while the number of lung cancer deaths was about 1.76 million, accounting for 18.4% of all cancer deaths [1]. Lung cancer also has the highest morbidity and mortality in China. According to the data released by China National Cancer Center in February 2018, the total incidence of lung cancer in 2014 was about 781,000, accounting for 20.6% of all cancer cases [2]. The mortality of lung cancer in China increased by 465% in the 30 years from 1983 to 2013 [3]. It is important to identify risk factors of lung cancer and to develop prevention policies involving all relevant areas.

There are many pathogenic factors for lung cancer. The recognized risk factors include smoking and passive smoking, family genetics, air pollution, work environment exposure and diet. Outdoor air pollution was identified as a cause of lung cancer by IARC under the World Health Organization (WHO) in 2013 [4]. With the rapid growth of motor vehicle traffic and rapid industrialization, the air pollution exposure of urban residents has significant effects on their respiratory health. According to the Global Burden of Disease, 3.2 million people died of air pollution in 2010, including 220,000 from lung cancer. Studies by the American Cancer Society (ACS) indicate that every 10 μg/m increase in PM_2.5_ increases lung cancer mortality by 8% [5].

The impact of built environment on public health is widely recognized [6,7,8]. From the perspective of urban planning, the “built environment” comprises urban design factors, land use, and the transportation system, and encompasses patterns of human activity within the physical environment [9]. Wang et al. [10] summarized four factors of built environment, including land use, spatial form, transportation system and green space which affect public health through two paths: (1) affecting air pollution and its human exposure; (2) affecting physical activity and communication. As far as lung cancer is concerned, a large number of studies have confirmed the impact of air pollution on the incidence of lung cancer, while it has also been confirmed that the arrangement of urban land use, transportation system, green space and other built environmental factors affect the distribution and diffusion of pollutants based on field examinations [11,12,13]. The scale and layout of specific types of urban land such as residential, green, industrial and water, etc. and its relevant human activities are significantly correlated with air quality [14,15]. A case-control study in UK found that years of residence near heavy industry (less than 5 kilometers) were associated with lung cancer. With a control group of inhabitants who lived near heavy industry for no more than one year, a relatively high standard incidence of lung cancer was discovered among residents who lived for more than 25 years [16]. Overall, compared with farm land, green space and waterbody, urban construction land, residential area, industrial area and mining have higher risk causing air pollution [17,18,19]. In terms of land use layout, the percentage of residential land patch and industrial land patch, the maximum patch index and patch fragmentation of construction land were significantly correlated with SO_2_ and NO_2_ concentration [20]. Studies have confirmed that the high risk of lung cancer incidence is associated with a short distance to main roads and high road density [21,22,23]. Traffic-related pollutants, such as nitrogen oxide, were discovered to exponentially decrease along with the distance from roads [24]. Therefore, changes in the urban built environment cause changes of air pollutant concentration at the micro-level, and then may affect the incidence of lung cancer.

A large number of studies have summarized the epidemiological characteristics of lung cancer, including age, sex and geographical context. Whether smokers or non-smokers, the risk of lung cancer increases with age [25]. The incidence of lung cancer is relatively rare under the age of 40. Above 40 years old, it rises rapidly, reaching its peak at around 70 years old [26]. Prospective cohort studies and available statistical data show that the incidence and mortality of lung cancer in men are higher than in women [27,28,29]. However, in the 21st century, the incidence of female lung cancer in developed countries has increased significantly. A meta-analysis of six cohort studies found that the incidence of lung cancer in women aged 40 to 79 who never smoked was slightly higher than that in men, although not significantly [30]. A recent study demonstrated a significant association between air pollution exposure and never-smokers compared to ever-smokers in women, and an association that was absent in males [31]. In China, the incidence of lung cancer varies in different geographical regions, especially between urban and rural areas [2]. Although epidemiological characteristics may not present as solid evidence of the pathogenic factors causing lung cancer, their significant impact on the health outcomes and spatial distribution of lung cancer has been confirmed.

Previous studies on the impact of the built environment on air pollution and the impact of air pollution on the incidence of lung cancer were relatively independent, which made it difficult to put forward specific interventions to promote respiratory health by improving the built environment. In addition, the built environment itself may have an indirect impact on respiratory health by affecting life style and working status [32,33]. Although the risk of disease varies individually, the interaction between genes and environment can best explain the incidence of disease, including cancer [34,35]. With time, the risk factors of the contextual built environment, natural environment and social environment, combined with biological factors in the human body, ultimately affect individual health. The improvement of the built environment may reduce the risk of disease in a larger population [36]. Studies have shown that some variables of built environments have significant effects on specific disease risks, such as heart disease, asthma and lung cancer [23,37]. It is worth further exploring the direct relationship between built environments and health outcomes.

This paper, therefore, explores the correlation between specific factors of the built environment and the incidence of lung cancer. The research questions in this paper are as follows: (1) does the spatial distribution of lung cancer patients have a regular pattern (or show certain spatial distribution pattern)? (2) Is there a correlation between lung cancer and the built environment? Based on the causal path of “built environment-outdoor air pollution-lung cancer” confirmed by previous studies, this study selects potential built environment variables that affect outdoor air pollution, and then uses spatial analysis method to explore the spatial distribution pattern of lung cancer and its correlation with built environment factors. Based on the identified significant built environmental factors, this paper proposes corresponding strategies for planning and design to promote respiratory heath as well as the development of healthy cities.

## 2. Materials and Methods

### 2.1. Study Site

**Figure 1:**
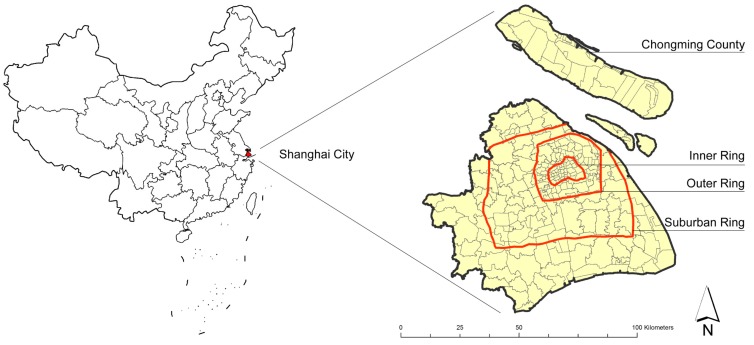
The location of the study site.

**Figure 2:**
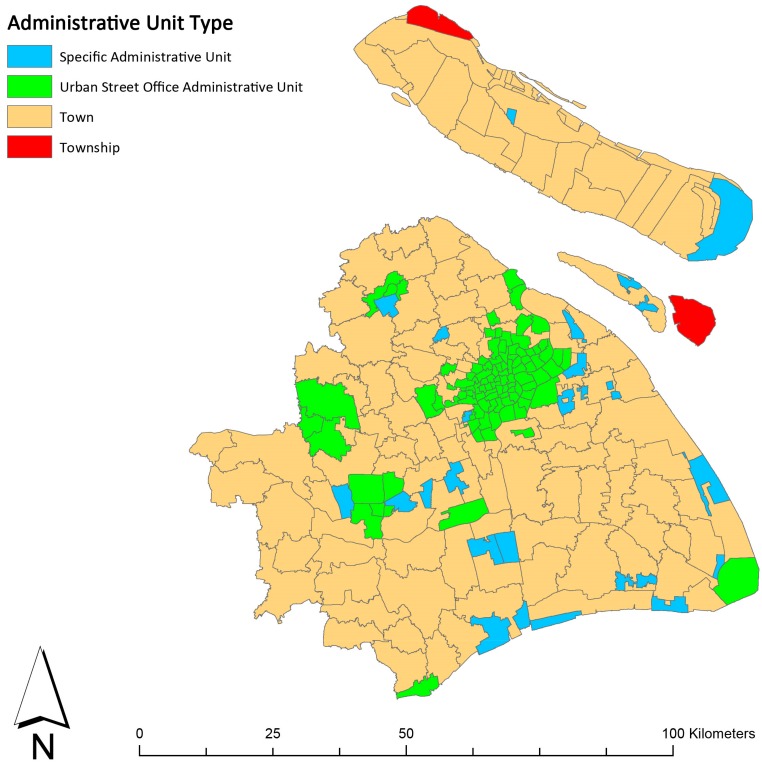
Administrative unit types in Shanghai.

This research selected Shanghai as the study site (Figure 1). Located in the eastern part of China, Shanghai is one of the four municipalities directly under the administrative control of the Central Government of China. Its total area is about 6340 square kilometers, with a total population of 24.18 million in 2017. In the Sixth National Population Census in 2010, there were 230 administrative units at the street/town/township level in Shanghai, comprised of 99 streets, 110 towns, 2 townships and 19 specific administrative units, including 5 industrial parks, 5 comprehensive technology development zones, 1 tourist area and 8 agricultural parks/farms (Figure 2). Street in Shanghai refers to a type of administrative unit within the urbanized area which is at the same administrative level as a town and township. The spatial unit analyzed in the study includes only four types: street, town, township and industrial park. Because some of the other types have been established after the registration of lung cancer cases or alternatively contain no residents, they were excluded from the analysis. The street/town/township level administrative unit is chosen as the basic spatial unit for data aggregation and analysis in cross-sectional research. The reasons are as follows: (1) Streets/towns/townships have large enough population bases to ensure that the calculation error of the incidence would be small, especially when calculating the age standardized incidence rate; (2) the function of the street/town/township administrative unit in Shanghai is relatively independent and complete, which can better reflect the overall characteristics of built environment. Shanghai usually could be divided into five regions according to the circular expressway: within the Inner Ring; the Inner Ring to the Outer Ring; the Outer Ring to the Suburban Ring (suburban areas); outside of the Suburban Ring (outer suburbs, except Chongming District); Chongming District (Figure 1). The central city area is inside the Outer Ring, while the suburbs are outside the Outer Ring. The study tracks the heterogeneity among these regions and takes street/town/township as the basic spatial unit for analysis.

### 2.2. Lung Cancer Data

Lung cancer data was provided by Shanghai Center for Disease Prevention and Control. The data includes sex, age, occupation, smoking history and family address information of lung cancer cases confirmed in all hospitals in Shanghai from 2009 to 2013. During this period, there were 53,805 new cases of lung cancer occurred in Shanghai, including 36,167 males (67.22%) and 17,638 females (32.78%).

Since most cohort studies show that lung cancer is rare in young people under 40 years of age, it would be difficult to measure the incidence of lung cancer in young people [25]. The population over 86 years old has a small base, so it is easy to cause calculation errors when calculating the incidence. The study, therefore, selects cases aged 50–86 for analysis, which accounted for 90% of the total number of cases. We excluded 1063 cases which could not be spatially located because of the lack of family address information. This process ended with 11,244 cases in the study, including 4634 males and 6610 females who never smoke and did not report passive smoking.

There exist several ways to measure the risk of lung cancer in a specific spatial unit. In this study, standardized incidence rate (SIR) is adopted to represent the risk of lung cancer. SIR can effectively avoid the influence of age structure on the measurement of lung cancer incidence comparing with raw rate. The formula for calculating the SIR of lung cancer is as follows:

(1) $$\mathrm{SIR}=\frac{\sum w_{i}R_{i}}{\sum w_{i}}$$

where SIR is the standardized incidence rate, $R_{i}$ is the age-specific incidence rate of the age group i, and $w_{i}$ is the weight of the age group i. The calculation of weight $w_{i}$ is as follows:

(2) $$w_{i}=\frac{N_{i}}{N}$$

where $N_{i}$ is the population of age group i in the standard population and N is the total population of the standard population. This study takes the population of the whole city as the standard population.

### 2.3. Environmental Factors of Lung Cancer and Their Proxies

**Figure 3:**
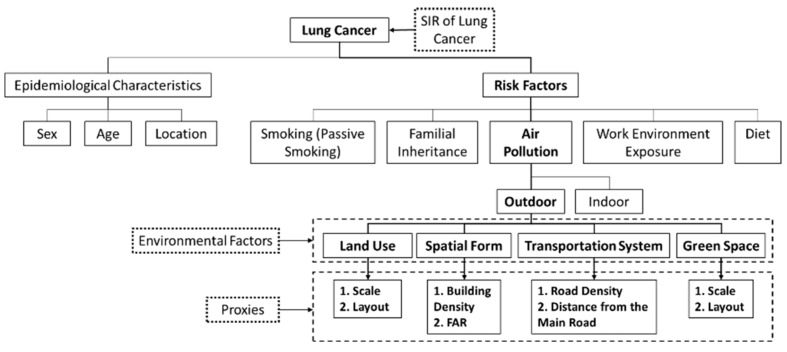
Risk factors of lung cancer and their proxies.

Epidemiological studies of lung cancer have identified risk factors for lung cancer such as smoking, passive smoking, familial inheritance, air pollution (outdoor and indoor air pollution), work environment exposure and diet [38]. Relevant studies have identified certain factors of urban built environment affecting outdoor air pollution, including land use, spatial form, transportation system, green space and public open space [39]. The scale and layout of specific types of land use may affect outdoor air pollution, thus affecting respiratory health, including industrial land [40,41,42,43,44], cultivated land [44], residential land [40,41,42,44] and waterbody [42,44]. Urban spatial form can affect the urban wind environment, thus affecting the emission and diffusion of air pollutants. The commonly used indicators reflecting urban spatial form are building density [41,45,46] and floor area ratio (FAR) [42]. Previous studies have confirmed the impact of transportation systems on the risk of lung cancer, involving the distance from the main roads and road density [21,22]. It is also confirmed that the scale and layout of green space have a significant impact on the atmospheric environment [47]. Figure 3 shows risk factors of lung cancer and their proxies.

This study, therefore, adopts the percentage of specific types of land to the total area of geographic units to reflect the characteristics of land use scale, and parcel density to reflect the characteristics of land use layout. More specifically, the greater the parcel density, the more dispersed the land use layout. The land use types for modeling include industrial, residential, commercial, cultivated, and rural developed land, as well as warehouse and land ready to be built. Under the title of this paper of built environments, we do include certain environmental factors outside the built area, such as cultivated land, in the analysis. The underlying reason is that the percentage and layout of cultivated land can reflect the proportion of primary industry and urban-rural characteristics at the street/town/township level. According to the percentage of cultivated land and other indicators, the entire city can be divided into rural areas, urban-rural mixed areas and urbanized areas. Variables of spatial form are not included in the study because building density and FAR cannot effectively represent the spatial form of suburbs. In terms of transportation system, this study uses the percentage of area for roads to total land and road density to reflect basic traffic volume within the street/town/township. The study differentiates the street/town/township with or without crossing expressways. For green land, this study uses the percentage of urban independent green space to the total area of street/town/township to measure its scale, and green space density to measure its layout feature.

Data for land use and road system was calculated through vectorization of Shanghai Land Use Status Map (2011) provided by Shanghai Municipal Bureau of Planning and Land Use. The population data was obtained from the sixth census in 2010 and the Point of Interest (POI) data was from Baidu Map.

### 2.4. Analytical Methods

With a focus on the spatial distribution pattern of lung cancer cases in Shanghai, this paper explores the correlation between built environment and health outcomes. The basic hypothesis is the following: (1) lung cancer cases are not randomly distributed in the city; (2) the spatial distribution of lung cancer cases in the city is related to the built environment. In order to verify these hypotheses, this study conducts two parts of research: (1) spatial autocorrelation analysis; (2) spatial stratified heterogeneity analysis.

For the first part of the research, the clustering pattern of lung cancer incidence is identified through the test of spatial autocorrelation. The regions with high, low and abnormal incidence are identified with this method. The spatial distribution pattern of lung cancer incidence is revealed, which provides clues for further attribution analysis. The analytic method includes Moran’s I and Local Indicators of Spatial Association (LISA) to explore the global and local spatial correlation of lung cancer in ArcGIS 10.3 analysis software (Esri, Redlands, CA, USA).

For the second part of the research, this study uses Geo-Detector to analyze the spatial stratified heterogeneity of the distribution of lung cancer cases. The Geo-Detector is based on the assumption that if an environmental factor plays an important role in a disease, the spatial distribution of the disease and environmental factors should present similar pattern [48,49]. Based on the variance analysis, the Geo-Detector uses Power of Determinant (PD) value to quantitatively measure the degree of disease explained by environmental factors. The formula for calculating the value of PD is as follows:

(3) $$\mathrm{PD}=1-\frac{\sum_{h=1}^{L}N_{h}\sigma_{h}^{2}}{N\sigma^{2}}$$

where *h* = 1,2,... *L* is the stratification of environmental factors. $N_{h}$ is the number of spatial units of the sub-region *h*, and N is the total number of spatial units of the whole region. $\sigma_{h}^{2}$ and $\sigma^{2}$ are the variances of the dependent variable within the sub-region h and the whole region respectively. The value of PD is between 0 and 1. The larger the value of PD, the higher the degree of interpretation of spatial distribution of disease by environmental factors.

Geo-Detector assumes a non-linear correlation between the environmental factors and the spatial distribution of diseases, and is designed to effectively avoid the multiple collinearity of independent variables in traditional regression models. A large number of studies have used Geo-Detector to analyze the relationship between disease distribution and built environment factors [50,51]. The detail steps of adopting Geo-Detector in this study is to discretize the built environment variables which have potential impacts on outdoor air pollution confirmed by relevant studies and then to establish various stratification with specific characteristics of built environment. Three modules of Geo-Detector are used including factor detector, risk detector and interaction detector. The factor detector aims to determinate the impact of built environment indicators on lung cancer incidence. The risk detector aims to analyze the effect of built environment variables on lung cancer incidence, while the interactive detector aims to analyze the interactive effects between built environment, population and economy.

## 3. Results

### 3.1. Descriptive Statistics of Lung Cancer Incidence and Built Environment Factors

**Figure 4:**
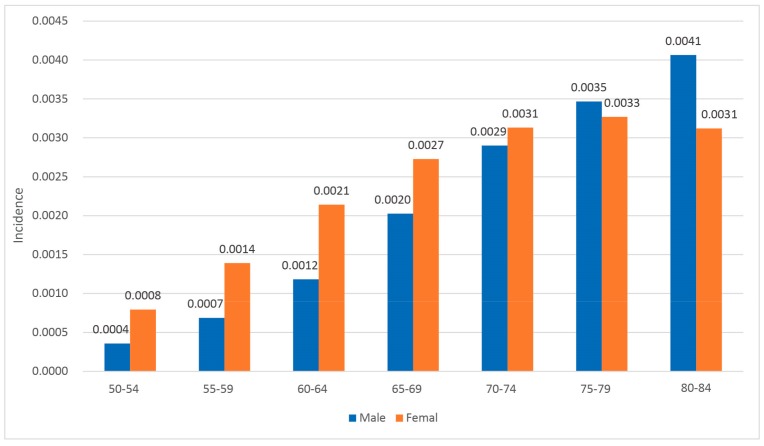
Incidence of lung cancer in male and female age groups.

The lowest SIR of street/town/township in Shanghai was zero, while the highest was 104.5/10,000 and the average was 7.86/10,000. The SIR of lung cancer in different sex and age groups presents different features (Figure 4). In most age groups, the SIR of lung cancer of women is higher than that of men, who never smoke and not report passive smoking. Contextual risk factors instead of smoking, therefore, might present a significant effect on women.

The distribution of the built environment factors varied across Shanghai (Table 1). The 75% quantile of most built environment variables differs greatly from its maximum value, which indicates that certain administrative units present abnormal values due to their specific functions. For example, while the average percentage value of industrial land is 8.88%, the maximum value is 52.69%, which suggesting the existing of master-planned industrial parks. While the average percentage of transportation land is 5.77%, the highest percentage of transportation land is 42.33%, which indicates there are large transport hubs in the specific administrative unit such as airports and railway stations. Although Geo-Detector can effectively deal with the problems caused by extreme values, it is still necessary to exclude special administrative units, in order to avoid the impact of extreme values on the analysis results.

### 3.2. Spatial Clustering Characteristics of Lung Cancer Cases

**Figure 5:**
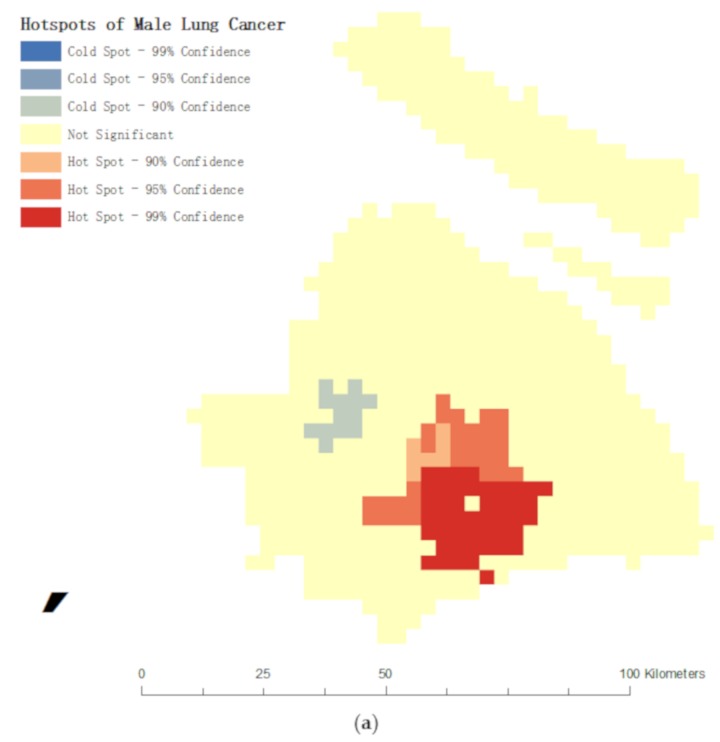

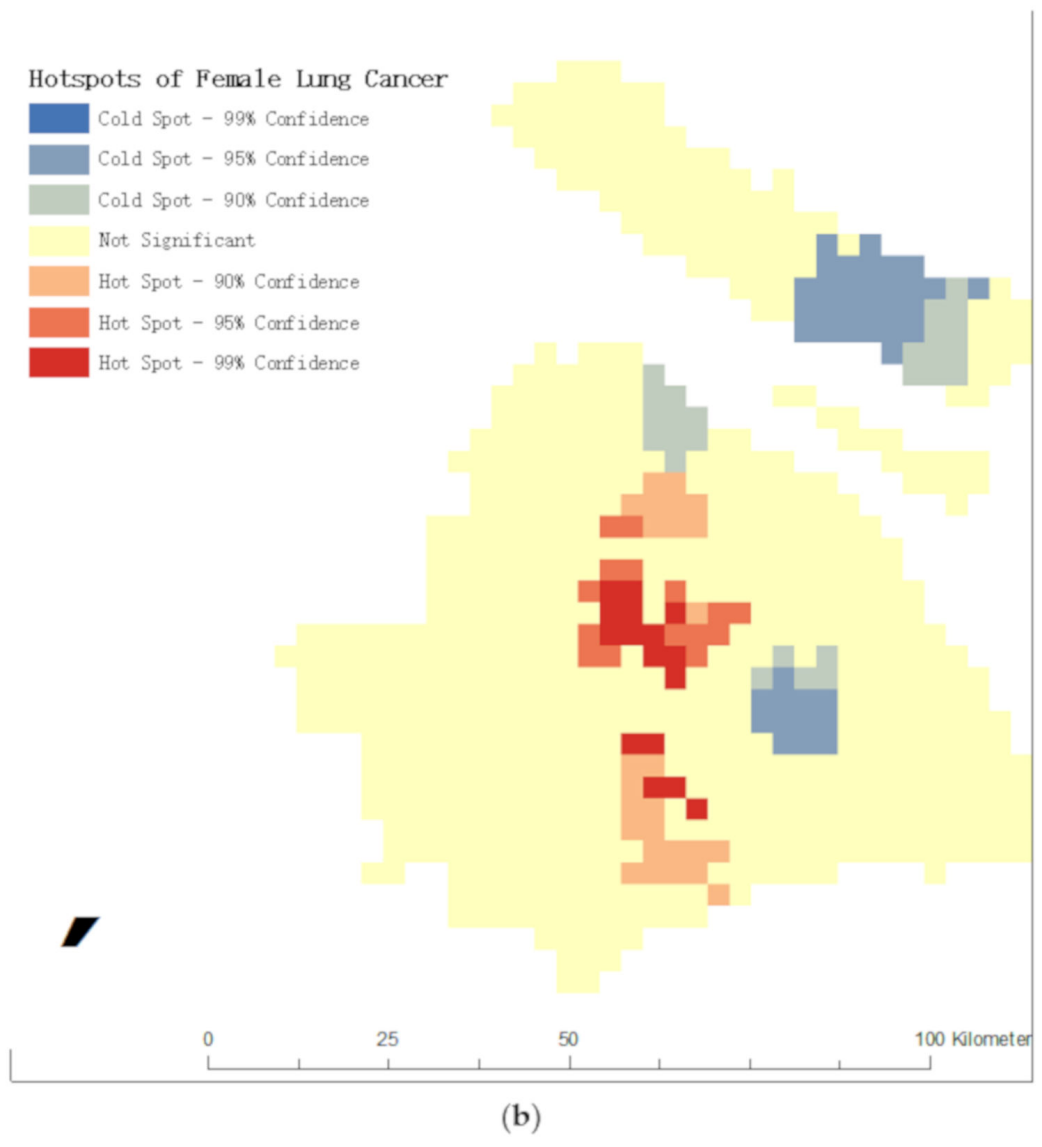
The result of hot spot analysis (**a**) Hotspots of male lung cancer; (**b**) Hotspots of female lung cancer.

The results of global spatial autocorrelation test show that Moran’s I and its significance are 0.039 (*p* = 0.001), 0.041 (*p* = 0.001) and 0.133 (*p* = 0.000) respectively for all residents, men and women. This means that the distribution of lung cancer cases in Shanghai presents a significant clustering feature. The spatial distribution of female lung cancer cases is higher than that of male lung cancer cases, which indicates that environmental factors may have a more significant impact on the incidence of female lung cancer. Hot spot analysis shows that high incidence areas of male and female lung cancer cases are located in several large-scale industrial parks (Figure 5). The results of LISA analysis show that the central urban area within the Inner Ring was also a relatively high incidence area of lung cancer for both men and women. This indicates that high-density urban areas may have a specific impact on the incidence of lung cancer.

### 3.3. Spatial Stratified Heterogeneity of Lung Cancer Cases

As described above, the factor detector of Geo-Detector is adopted to unravel the impact of risk factors on the incidence of lung cancer, and the variables ranked by PD value as follows: administrative unit type (0.64) > industrial land percentage (0.28) > cultivated land percentage (0.06) and green land percentage (0.05) (Table 2). The result shows that the type of administrative units and the percentage of industrial land are the two main factors to explain the spatial stratified heterogeneity of lung cancer. The percentage of cultivated land and green land can also explain the distribution of lung cancer to a certain extent.

The risk detector is adopted to analyze the detailed effect of these variables on the SIR of lung cancer. Table 3 shows the effect of the type of administrative unit measured by urbanization rate on the lung cancer incidence. With the increase of urbanization rate, the average SIR of lung cancer in towns (4.21/10,000), townships (6.86/10,000) and streets (7.29/10,000) gradually increased. The average SIR of lung cancer in industrial parks (63.52/10,000) was significantly higher, about 10 times the incidence of other administrative units. The results are consistent with the results of hot spot analysis, indicating that industrial parks are high-risk areas for lung cancer.

Table 4 shows the impact of the percentage of industrial land on the incidence of lung cancer. When the percentage of industrial land exceeds 30%, the SIR of lung cancer increases significantly. There is no significant difference in lung cancer incidence between regions with less than 10% of industrial land and regions with 10% to 30% of industrial land. As for the percentage of cultivated land and green land, there is no significant difference in the risk of lung cancer when respectively increasing the value of these two variables.

It has been found that the risk of lung cancer in the administrative areas with large-scale industrial park is high. In order to avoid the possible extreme value impact of industrial parks, factor detectors and risk detectors of Geo-Detector are repeatedly used after excluding the variable of industrial park. The new results show that the spatial stratified heterogeneity of lung cancer incidence caused by the administrative unit type is no longer significant, and the scale and layout of industrial land is no longer significant as well. Combining with the consideration of industrial park locations, it indicates that the impact of industrial land on lung cancer is mainly located in industrial parks, especially in the suburbs, but not in other areas. Location presents a significant correlation with the distribution of lung cancer in the new results, which can explain the incidence and distribution of lung cancer to 10.8%.

For representing the location, five regions are classified according to the important traffic routes in Shanghai. Among them, the highest incidence of lung cancer is found in the areas within the Inner Ring and the Outer Suburbs, which is 8.08/10,000 and 7.81/10,000, respectively, followed by the areas from the Inner Ring to the Outer Ring and suburban areas, where the incidence of lung cancer is 6.56/10,000 and 6.69/10,000, respectively. The incidence of lung cancer in Chongming District is the lowest, 5.00/10,000.

The analysis results after excluding industrial parks show that the explanatory degree of the percentage of cultivated land to lung cancer incidence increases to 11.3%. The correlation between the percentage of cultivated land and the incidence of lung cancer is inverted “U” type. The average incidence of lung cancer is lower in areas with cultivated land area accounting for more than 60% or less than 1%, while the average incidence of lung cancer is higher in areas with cultivated land area accounting for 20–60%. This indicates that the incidence of the outer suburban area with an urban-rural mixture is higher than that of urban and rural areas.

**Figure 6:**
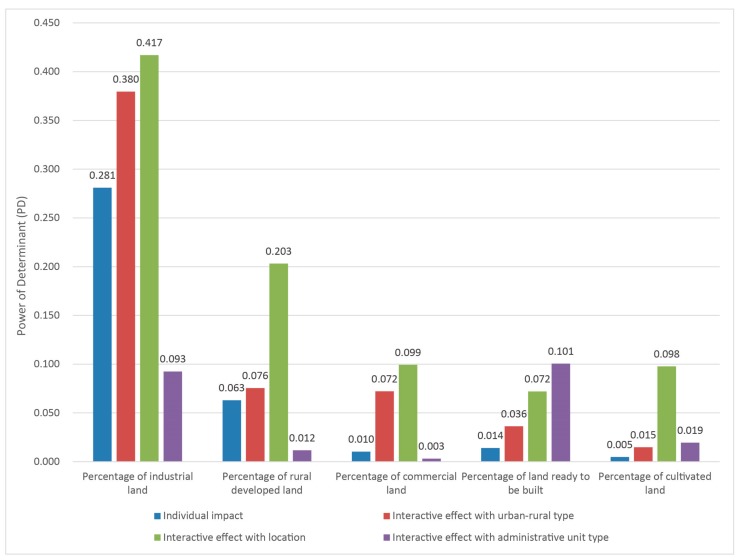
Interactive impact of percentage of land use and urban-rural type, location and administrative unit type on lung cancer.

Finally, we use the interactive detector of Geo-Detector to analyze the interaction of built environmental variables on the incidence of lung cancer. Figure 6 shows the interaction between certain land uses and urban-rural types, locations and administrative unit types. The results show that the PD value of land use variables significantly increases after the interaction with urban and rural types and locations, which indicates that there is a synergistic effect between land use variables, urban and rural types and locations. Furthermore, the interaction between land use percentage and land distribution has a higher degree of explanation for lung cancer incidence than its respective effect.

## 4. Discussion

Clustering and geographic correlation are two significant approaches in spatial epidemiology [52]. This study, therefore, analyzes the spatial autocorrelation and stratified heterogeneity of lung cancer cases, so as to explore the geographic correlation between lung cancer incidence and built environment factors. Arguably one of the biggest challenges facing spatial epidemiology is that of identifying geographic distribution pattern (e.g., outliers, clusters) above and beyond background variation [53], based on which this study develops two hypotheses to test. The first hypothesis is confirmed by the result of clustering analysis: the spatial distribution of male and female lung cancer cases in Shanghai presents significant clustering characteristics, with a more obvious pattern of female lung cancer cases. There are two types of high incidence areas of lung cancer: one located near large industrial parks and the other located in urban central areas. The second hypothesis has been verified that the spatial distribution of lung cancer cases is correlated with the types of administrative units, the geographical location of cases, land use and green space.

Our study found that the type of administrative unit could explain 64.3% of the spatial distribution of lung cancer cases. The classification of urban administrative units is mainly based on the level of economic development and economic structure, reflecting the basic functions and characteristics of a spatial unit. As one of the specific types of administrative unit in the study, industrial parks consist of a large scale of industrial land, accounting for 30–50% of the total land use, and the layout of industrial land is relatively concentrated. Usually around 10% of urban residential land, 5% of rural construction land and 15% of farm land is located in these industrial parks. This study found that residents living within the administrative unit labeled as industrial park have a significantly high risk of respiratory health. Our findings for industrial pollution affecting lung cancer are consistent with previous studies, including a review reporting in 1990 that people living near non-ferrous smelters and a variety of other heavy industrial types may have an increasing risk of lung cancer in in Italy and Spain [54]. We don’t find that industrial land has a significant impact on lung cancer in areas other than industrial parks, possibly because the areas of other industrial land are smaller and then have a slim influence on public health. It may also because their industry types have no air pollution.

When industrial parks are excluded from the analysis, we find that geographical location is the most important environmental factor to explain the spatial distribution of lung cancer. It is defined according to the important traffic routes. The administrative units in the same geographical location not only have similar built environment characteristics, but also have more similar function orientations and economic structures.

The results of this study support that the incidence of lung cancer in rural areas is lower than that in urban areas, which was proposed by the latest epidemiological study of lung cancer in China as the incidence of lung cancer in Chongming District is significantly lower than other areas in Shanghai [55]. However, in some similar studies, the classification methods of urban and rural areas are single and dualistic, and the areas between urban and rural areas are neglected. This study discovers that the risk in outer suburbs presents as significantly higher than that in urban peripheries. Outer suburbs present a typical urban-rural mixture with average cultivated land that accounts for 50% of total land use, while urban peripheries have mostly completed urbanization (average cultivated land accounts for 26%). This is consistent with the results of risk detector analysis. Compared with urban and rural areas, respiratory health risk in areas with urban-rural mixture is higher.

Our study also found that urban areas within the inner ring present a high risk for lung cancer, which is consistent with the results of local spatial autocorrelation analysis. A similar study showed that the relative risk of lung cancer for residents living in the city center was 1.5 (95% CI: 1.0–2.2) compared with ordinary residents, possibly due to excessive air pollution [56]. Since the high-density urban area within the Inner Ring in Shanghai does not contain industrial land, the reason for its high incidence of lung cancer may be traffic and other pollution factors, which needs further exploration.

The result of interaction detector analysis shows that urban-rural types and locations play a synergistic role in the impact of built environment on the incidence, which indicates that the impact of built environment on respiratory health presents different between urban and rural areas as well as in different locations. The spatial distribution of lung cancer is affected by both the scale and layout of land use. The interaction analysis shows that the impact of one built environment factor on lung cancer is adjusted and enhanced by other built environment factors.

There are two main limitations in this study. First, the impact of workplace exposure on the incidence of lung cancer has not been included in the analysis due to the lack of data. Secondly, this study does not include the impacts of micro-community environment and indoor residential environment. Future research can capture these impacts with variables to represent spatial features of these two levels.

## 5. Conclusions

The main finding of this study is that large industrial parks and their adjacent areas, high-density urban central areas and outer suburban areas, are high incidence areas of lung cancer in Shanghai. Based on the significant variables identified by the Geo-Detector, suggestions for planning intervention include: (1) protective green belts need to be enhanced in order to eliminate the potential pollution of large-scale industrial parks; (2) better environment regulation may be required in the outer suburban areas; (3) urban areas with high-density of buildings and roads need better solutions for decreasing air pollution from heavy traffic.

This cross-sectional study attempts to identify potential built environment factors associated with lung cancer, while it cannot reveal the temporal relationship between environmental exposure and health outcomes. Further study is needed to discover the causality between built environment and lung cancer. More specific areas such as suburbs or urban centers can be respectively and thoroughly explored in the further study. Longitudinal rather than cross-sectional studies can be used to unravel the causal relationship between built environmental factors and respiratory health outcomes.

## Figures and Tables

**Table 1:** Distribution of SIR of lung cancer and built environment variables.

Variables		Mean	Minimum	25%	50%	75%	Maximum
SIR ^1^ (new cases/10,000)		7.86	0.00	4.56	6.63	8.90	104.5
Land use	Percentage of industrial land	8.88	0.00	2.11	5.87	12.21	52.69
	Industrial parcel density (#/km^2^)	113.02	0.00	34.79	53.73	99.51	1699.15
	Percentage of high-quality residential land	13.62	0.00	0.85	7.08	25.06	56.15
	High-quality residential parcel density (#/km^2^)	54.31	0.00	22.29	32.37	55.47	638.16
	Percentage of Low-quality residential land	1.52	0.00	0.05	0.46	1.23	21.37
	Low-quality residential parcel density (#/km^2^)	113.59	0.00	31.72	64.41	130.26	1276.32
	Percentage of rural developed land	4.52	0.00	0.00	1.63	8.33	20.83
	Developed parcel density in rural area (#/km^2^)	118.18	0.00	0.00	78.60	122.43	1215.28
	Percentage of commercial land	1.88	0.00	0.32	1.20	2.82	12.19
	Commercial parcel density (#/km^2^)	175.24	0.00	80.39	138.02	220.25	1391.98
	Percentage of land ready to be built	5.71	0.00	1.76	4.53	7.99	43.20
	Density of parcel ready to be built (#/km^2^)	74.80	0.00	35.35	55.44	95.33	431.22
	Percentage of cultivated land	24.36	0.00	0.00	12.16	49.39	86.23
	Cultivated land density (#/km^2^)	33.39	0.00	0.00	6.86	17.95	1755.62
Transportation system	Road density (#/km^2^)	6.41	0.33	3.06	5.68	9.19	22.78
	Main road density (#/km^2^)	0.41	0.00	0.11	0.28	0.59	2.11
	Percentage of transportation land	5.77	0.03	2.63	5.26	6.99	42.33
Green space	Percentage of green space	2.79	0.00	0.57	1.65	3.60	70.92
	Green space density (#/km^2^)	199.78	0.00	92.32	171.28	264.67	920.37

^1^ SIR refers to standardized incidence rate. # refers to number of parcels.

**Table 2:** Spatial association between built environment factors and lung cancer incidence.

Variables		All Space Units Included		Industrial Parks Excluded	
		PD^3^	*p*-Value	PD	*p*-Value
Land use	Administrative unit type ^1^	0.64	0.000 ***	0.02	0.745
	Location ^2^	0.04	0.08	0.11	0.004 **
	Percentage of industrial land	0.28	0.000 ***	0.04	0.978
	Industrial parcel density (#/km^2^)	0.01	0.909	0.07	0.263
	Percentage of high-quality residential land	0.01	0.263	0.02	0.556
	High-quality residential parcel density (#/km^2^)	0.00	/	0.05	0.622
	Percentage of Low-quality residential land	0.02	0.843	0.07	0.355
	Low-quality residential parcel density (#/km^2^)	0.00	/	0.00	/
	Percentage of rural developed land	0.01	0.362	0.02	0.471
	Developed parcel density in rural area (#/km^2^)	0.03	0.370	0.03	0.935
	Percentage of commercial land	0.01	0.666	0.02	0.988
	Commercial parcel density (#/km^2^)	0.01	0.489	0.02	0.915
	Percentage of land ready to be built	0.01	0.365	0.01	0.564
	Density of parcel ready to be built (#/km^2^)	0.02	0.548	0.01	0.976
	Percentage of cultivated land	0.06	0.03 *	0.11	0.027 *
	Cultivated land density (#/km^2^)	0.01	0.259	0.02	0.871
Transportation system	Road density (#/km^2^)	0.01	0.547	0.01	0.987
	Main road density (#/km^2^)	0.01	0.566	0.03	0.500
	Percentage of transportation land	0.02	0.261	0.01	0.986
	With or without crossing expressway	0.00	/	0.00	/
Green space	Percentage of green space	0.05	0.037 *	0.03	0.452
	Green space density (#/km^2^)	0.01	0.573	0.11	0.318

^1^ Administrative unit type refers to street, town, township and industrial park; ^2^ Location refers to five regions divided by the circular expressway: within the Inner Ring; the Inner Ring to the Outer Ring; the Outer Ring to the Suburban Ring (suburban areas); outside of the Suburban Ring (outer suburbs, except Chongming District); Chongming District. ^3^ PD refers to Power of Determinant. # refers to number of parcels. * *p* < 0.05, ** *p* < 0.01, *** *p* < 0.001.

**Table 3:** SIR of lung cancer according to the administrative unit type stratum.

Stratum	Street	Town	Township	Industrial Park
SIR (cases/10,000)	7.29	6.86	4.21	63.52

**Table 4:** SIR of lung cancer according to the percentage of industrial land stratum.

Stratum	<10%	10–30%	>30%
SIR (cases/10,000)	7.05	7.46	31.59

## References

[1] Bray F., Ferlay J., Soerjomataram I., Siegel R.L., Torre L.A., Jemal A. (2018). Global cancer statistics 2018: GLOBOCAN estimates of incidence and mortality worldwide for 36 cancers in 185 countries. CA Cancer J. Clin..

[2] Chen W., Sun K., Zheng R., Zeng H., Zhang S., Xia C., Yang Z., Li H., Zou X., He J. (2018). Cancer incidence and mortality in China, 2014. Chin. J. Cancer Res..

[3] Wang Y.-C., Wei L.-J., Liu J.-T., Li S.-X., Wang Q.-S. (2012). Comparison of cancer incidence between China and the USA. Cancer Biol. Med..

[4] Loomis D., Grosse Y., Lauby-Secretan B., Ghissassi F.E., Bouvard V., Benbrahim-Tallaa L., Guha N., Baan R., Mattock H., Straif K. (2013). The carcinogenicity of outdoor air pollution. Lancet Oncol..

[5] Pope C.A. III, Burnett R.T., Thun M.J., Calle E.E., Daniel K., Kazuhiko I., Thurston G.D. (2002). Lung cancer, cardiopulmonary mortality, and long-term exposure to fine particulate air pollution. JAMA.

[6] Perdue W.C., Stone L.A., Gostin L.O. (2003). The built environment and its relationship to the public’s health: The legal framework. Am. J. Public Health.

[7] Evans G.W. (2003). The built environment and mental health. J. Urban Health-Bull. N. Y. Acad. Med..

[8] Srinivasan S., O’Fallon L.A. (2003). Creating healthy communities, healthy homes, healthy people: Initiating a research agenda on the built environment and public health. Am. J. Public Health.

[9] Handy S.L., Boarnet M.G., Ewing R., Killingsworth R.E. (2002). How the built environment affects physical activity: Views from urban planning. Am. J. Prev. Med..

[10] Lan W., Shuwen L., Xiaojing Z. (2018). Exploration of approaches and factors for healthy city planning. China City Planning Review.

[11] Xu H., Bi X.H., Zheng W.W., Wu J.H., Feng Y.C. (2015). Particulate matter mass and chemical component concentrations over four Chinese cities along the western Pacific coast. Environ. Sci. Pollut. Res. Int..

[12] Weng Q., Yang S. (2006). Urban air pollution patterns, land Use, and thermal landscape: An examination of the linkage using GIS. Environ. Monit. Assess..

[13] Schweitzer L., Zhou J. (2010). Neighborhood air quality, respiratory health, and vulnerable populations in compact and sprawled regions. J. Am. Plan. Assoc..

[14] Durstine J.L., Gordon B., Wang Z., Luo X. (2013). Chronic disease and the link to physical activity. J. Sport Health Sci..

[15] Mackenbach J.D., Rutter H., Compernolle S., Glonti K., Oppert J.M., Charreire H., De B.I., Brug J., Nijpels G., Lakerveld J. (2014). Obesogenic environments: A systematic review of the association between the physical environment and adult weight status, the SPOTLIGHT project. BMC Public Health.

[16] Edwards R., Plessmulloli T., Howel D., Chadwick T., Bhopal R., Harrison R., Gribbin H. (2006). Does living near heavy industry cause lung cancer in women? A case-control study using life grid interviews. Thorax.

[17] Tang X., Liu H., Li J., Xie Z., Zhao W. (2015). Response analysis of haze/particulate matter pollution to land use/cover in Beijing. China Environ. Sci..

[18] Xu S., Zou B., Pu Q., Guo Y. (2015). Impact analysis of land use/cover on air pollution. J. Geo-Inf. Sci..

[19] Shi Y., Li R., Qiu J., Huang D., Wang H. (2017). Spatial distribution simulation and underlying surface factors analysis of NO2 concentration based on land use regression spatial distribution simulation and underlying surface factors analysis of NO_2_ concentration based on land use regression. J. Geo-Inf. Sci..

[20] Cui Y. (2013). Research of the Influence City Land Use Change on Air Environment Quality.

[21] Gilescorti B., Vernezmoudon A., Reis R., Turrell G., Dannenberg A.L., Badland H., Foster S., Lowe M., Sallis J.F., Stevenson M. (2016). City planning and population health: A global challenge. Lancet.

[22] Zhu Y., Hinds W.C., Kim S., Sioutas C. (2002). Concentration and size distribution of ultrafine particles near a major highway. J. Air Waste Manag. Assoc..

[23] Chen F., Jackson H., Bina W.F. (2009). Lung adenocarcinoma incidence rates and their relation to motor vehicle density. Cancer Epidemiol. Biomark Prev..

[24] De H.K., Korek M., Vienneau D., Keuken M., Kukkonen J., Nieuwenhuijsen M.J., Badaloni C., Beelen R., Bolignano A., Cesaroni G. (2014). Comparing land use regression and dispersion modelling to assess residential exposure to ambient air pollution for epidemiological studies. Environ. Int..

[25] Samet J.M., Avila-Tang E., Boffetta P., Hannan L.M., Olivo-Marston S., Thun M.J., Rudin C.M. (2009). Lung cancer in never smokers: Clinical epidemiology and environmental risk factors. Clin. Cancer Res..

[26] Thun M.J., Henley S.J., Burns D., Jemal A., Shanks T.G., Calle E.E. (2006). Lung cancer death rates in lifelong nonsmokers. J. Natl. Cancer Inst..

[27] Thun M.J., Henley S.J., Calle E.E. (2002). Tobacco use and cancer: An epidemiologic perspective for geneticists. Oncogene.

[28] Prescott E., Osler M., Hein H.O., Borchjohnsen K., Lange P., Schnohr P., Vestbo J. (1998). Gender and smoking-related risk of lung cancer. The Copenhagen center for prospective population studies. Epidemiology.

[29] Boffetta P., Clark S., Min S., Gislefoss R., Peto R., Andersen A. (2006). Serum cotinine level as predictor of lung cancer risk. Cancer Epidemiol. Biomark. Prev..

[30] Wakelee H.A., Chang E.T., Gomez S.L., Keegan T.H., Feskanich D., Clarke C.A., Holmberg L., Yong L.C., Kolonel L.N., Gould M.K. (2007). Lung cancer incidence in never smokers. J. Clin. Oncol..

[31] Myers R., Brauer M., Ladhar S., Atkar-Khattra S., Yee J., Ho C., Mcguire A., Grant K., Lee A., Melosky B. (2018). OA09. 07 Association between outdoor air pollution and lung cancer in female never smokers. J. Thoracic Oncol..

[32] Booth K.M., Pinkston M.M., Poston W.S.C. (2005). Obesity and the built environment. J. Am. Diet. Assoc..

[33] Brownson R.C., Hoehner C.M., Day K., Forsyth A., Sallis J.F. (2009). Measuring the built environment for physical activity: State of the science. Am. J. Prev. Med..

[34] Vandenbroucke J.P. (1988). Is ‘The Causes of Cancer’ a Miasma theory for the end of the twentieth century?. Int. J. Epidemiol..

[35] Wu S., Powers S., Zhu W., Hannun Y.A. (2016). Substantial contribution of extrinsic risk factors to cancer development. Nature.

[36] Sarkar C., Webster C., Gallacher J. (2014). Healthy Cities: Public Health through Urban Planning.

[37] Mitchell R., Popham F. (2008). Effect of exposure to natural environment on health inequalities: An observational population study. Lancet.

[38] Gazdar A.F., Zhou C., Pass H.I., Ball D., Scagliotti G.V. (2018). 4 - Lung Cancer in Never-Smokers: A Different Disease. IASLC Thoracic Oncology.

[39] Wang L., Zhao X., Xu W., Tang J., Jiang X. (2016). Correlation analysis of lung cancer and urban spatial factor: Based on survey in Shanghai. J. Thorac. Dis..

[40] Choe S.-A., Kauderer S., Eliot M.N., Glazer K.B., Kingsley S.L., Carlson L., Awad Y.A., Schwartz J.D., Savitz D.A., Wellenius G.A. (2018). Air pollution, land use, and complications of pregnancy. Sci. Total Environ..

[41] Son Y., Osornio-Vargas Á.R., O’Neill M.S., Hystad P., Texcalac-Sangrador J.L., Ohman-Strickland P., Meng Q., Schwander S. (2018). Land use regression models to assess air pollution exposure in Mexico City using finer spatial and temporal input parameters. Sci. Total Environ..

[42] Bertazzon S., Johnson M., Eccles K., Kaplan G.G. (2015). Accounting for spatial effects in land use regression for urban air pollution modeling. Spat. Spatio-Temporal Epidemiol..

[43] Habermann M., Billger M., Haeger-Eugensson M. (2015). Land use regression as method to model air pollution. previous results for Gothenburg/Sweden. Procedia Eng..

[44] Naughton O., Donnelly A., Nolan P., Pilla F., Misstear B.D., Broderick B. (2018). A land use regression model for explaining spatial variation in air pollution levels using a wind sector based approach. Sci. Total Environ..

[45] Li C., Wang Z., Li B., Peng Z.-R., Fu Q. (2018). Investigating the relationship between air pollution variation and urban form. Build. Environ..

[46] Shi Y., Xie X., Fung J.C.-H., Ng E. (2018). Identifying critical building morphological design factors of street-level air pollution dispersion in high-density built environment using mobile monitoring. Build. Environ..

[47] Coppel G., Wüstemann H. (2017). The impact of urban green space on health in Berlin, Germany: Empirical findings and implications for urban planning. Landsc. Urban Plan.

[48] Wang J.F., Li X.H., Christakos G., Liao Y.L., Zhang T., Gu X., Zheng X.Y. (2010). Geographical detectors-based health risk assessment and its application in the neural tube defects study of the Heshun Region, China. Int. J. Geogr. Inf. Sci..

[49] Wang J.-F., Zhang T.-L., Fu B.-J. (2016). A measure of spatial stratified heterogeneity. Ecol. Indic..

[50] Liao Y., Wang J., Wu J., Driskell L., Wang W., Zhang T., Xue G., Zheng X. (2010). Spatial analysis of neural tube defects in a rural coal mining area. Int. J. Environ. Health Res..

[51] Huang J., Wang J., Bo Y., Xu C., Hu M., Huang D. (2014). Identification of health risks of hand, foot and mouth disease in China using the geographical detector technique. Int. J. Environ. Res. Public Health.

[52] Elliott P., Wartenberg D. (2004). Spatial epidemiology: Current approaches and future challenges. Environ. Health Perspect..

[53] Goovaerts P., Jacquez G.M. (2004). Accounting for regional background and population size in the detection of spatial clusters and outliers using geostatistical filtering and spatial neutral models: The case of lung cancer in Long Island, New York. Int. J. Health Geogr..

[54] Benedetti M., Lavarone I., Comba P. (2001). Cancer risk associated with residential proximity to industrial sites: A review. Arch. Environ. Health.

[55] Chen W., Zheng R., Zeng H., Zhang S. (2015). Epidemiology of lung cancer in China. Thorac. Cancer.

[56] Barbone F., Bovenzi M., Cavallieri F., Stanta G. (1995). Air pollution and lung cancer in Trieste, Italy. Am. J. Epidemiol..

